# Parasites: From Source to Vector and Human

**DOI:** 10.1155/2014/780715

**Published:** 2014-12-21

**Authors:** Veeranoot Nissapatorn, Nongyao Sawangjaroen, Rogan Lee, Subhash Chandra Parija

**Affiliations:** ^1^Department of Parasitology, Faculty of Medicine, University of Malaya, 50603 Kuala Lumpur, Malaysia; ^2^Department of Microbiology, Faculty of Science, Prince of Songkla University, Hat Yai, Songkhla 90112, Thailand; ^3^Institute of Clinical Pathology and Medical Research (ICPMR), Westmead Hospital, Sydney, NSW 2145, Australia; ^4^Department of Microbiology, Jawaharlal Institute of Postgraduate Medical Education & Research (JIPMER), Pondicherry 605006, India

Parasites cause a significant burden of disease globally and the majority of parasitic infections fall into the category of Neglected Tropical Diseases (NTDs). The emergence of parasitic diseases, including zoonotic infections, is associated with the process of rapid urbanization, exponential population growth, borderless trade between countries and climate change. To adopt new methods of control, a number of important issues must be addressed such as environmental-based interventions, novel drug discovery, the next generation sero-markers and development of new strategies for parasite control, and so forth. In this special issue on parasites: from source to vector and human, we have invited a few papers that address such issues.

The first paper of this special issue addresses serine proteinase activities in excretory-secretory products of* Toxocara canis* second stage larvae (TES) related to the known sequences of TES components for example, TES26 and MUC4. The authors use protein modelling to identify molecules that can be incorporated into diagnostic and therapeutic techniques ultimately leading to better methods of control. The second paper on Chagas' disease describes extensively on aspects of transmission, association with pregnant women and congenital infection, clinical manifestations, diagnosis, treatment, control strategies, case follow-up and disease complications in Latin America.

The third paper of this special issue presents a brief overview of the helminth-associated immune response, the currently available helminth secretome data, some major secretome-derived immunomodulatory molecules, their potential mode of actions and the applicability of helminth-derived therapeutic proteins in the treatment of allergic and autoimmune inflammatory disease. The fourth paper addresses the greater risk of exposure to tick borne pathogens (*Anaplasma phagocytophilum* and* Ehrlichia chaffeensis*) among rural residents using serological mass screening in China. Significant risk factors associated with infection are: having contact with animals, planting crops, having more employment time and having a history of fever. This study also proposes safety measures required to reduce zoonotic transmission.

The fifth paper shows the effectiveness of different concentrations of two fractions of Curcuma longa cortex rich in turmerones and their respective liposomal formulations for treating promastigote forms of* Leishmania amazonensis*. The hexane fraction from the turmeric cortex, incorporated in liposomal formulation (LipoRHIC), presents a promising result for a new anti-leishmanial agent. The sixth paper uses climate modelling for global warming to indicate geographical spread, increasing number of seasonal generations and epidemiological changes of* Dirofilaria* spp. In addition to dirofilariosis, it also covers other mosquito-borne diseases in Russia and its neighbouring countries. The final paper of this special issue reports on the next generation sero-marker for toxoplasmosis with improved specificity and sensitivity using* Toxoplasma gondii* dense granular protein-5 (GRA5) on human serum samples.

*Veeranoot Nissapatorn* *Nongyao Sawangjaroen* *Rogan Lee* *Subhash Chandra Parija*

